# Dual pathways of aging stereotype threat at work: impacts on work behaviors of older workers

**DOI:** 10.3389/fpsyg.2025.1486911

**Published:** 2025-01-30

**Authors:** Lidan Liu, Zhongjun Wang, Xicheng Guo, Sulei Li, Xiaodi Wu

**Affiliations:** ^1^Department of Applied Psychology, Hubei University of Chinese Medicine, Wuhan, China; ^2^School of Psychology, Central China Normal University, Wuhan, Hubei, China

**Keywords:** aging stereotype threat, aging identity, psychological reactance, work withdrawal behavior, proactive work behavior, self-efficacy

## Abstract

Aging stereotype threat is a significant issue in modern workplaces, affecting older workers’ self-perceptions and work behaviors. Although research often highlights the negative impacts of aging stereotypes, the dual pathways by which these stereotypes influence negative and positive work behaviors remain underexplored. This study aims to address this gap by employing a dual mediation model, grounded in Uncertainty-Identity Theory and Psychological Reactance Theory, to explore the effects of aging stereotype threat on older workers’ behaviors. Using structural equation modeling (SEM) with two-wave data from older employees in various industries, the study shows that aging stereotype threat significantly affects aging identity, which fully mediates its link to work withdrawal behaviors. In contrast, psychological reactance emerges as another mediator, leading to proactive work behaviors. The results also suggest that older workers with high self-efficacy exhibit a stronger relationship between stereotype threat and aging identity. This research adds to the literature by exploring how aging stereotype threat leads to both negative and positive behavioral outcomes. It provides valuable insights for organizations aiming to support older employees in the workplace.

## Introduction

The aging workforce has become a focal point of research as global demographics shift towards older populations. As organizations increasingly rely on the expertise and experience of older workers, understanding the psychological and behavioral dynamics of this demographic becomes essential. One critical area of concern is the impact of aging stereotype threat—anxiety and concern experienced by older workers when they perceive negative stereotypes related to aging in the workplace (Steele and Aronson, 1995; Kalokerinos et al., 2014; von Hippel et al., 2019). Existing research on age-related stereotypes mainly emphasizes their negative effects on work attitudes, motivation, and well-being (Cheung et al., 2016; Desmette and Gaillard, 2008; Manzi et al., 2019; von Hippel et al., 2013; von Hippel et al., 2019). However, the nuanced ways in which aging stereotype threat influences specific work behaviors, both negative and positive, remain underexplored.

Historically, studies on stereotype threat have primarily centered on race and gender, illustrating how negative stereotypes can lead to decreased performance and disengagement (Steele et al., 2002). For older workers, the stereotype of declining competence, unwillingness to learn and resistance to change can be particularly damaging, potentially leading to work withdrawal behaviors, such as reduced effort, absenteeism, and even early retirement (Barber and Lui, 2020; Gringart et al., 2013; Schmader et al., 2001). These behaviors not only harm the individuals but also impose significant costs on organizations (Mathieu and Kohler, 1990). While social identity theory has traditionally provided a useful framework for understanding these dynamics, suggesting that when individuals perceive their social group as devalued, they may disengage to protect their self-esteem (Westberg et al., 2020), this study proposes an integrated approach. In the context of aging, Uncertainty-Identity Theory (Hogg, 2012) helps explain how the perception of negative stereotypes leads to a greater reliance on group identity to reduce uncertainty. For instance, older workers may identify more strongly with their age group to reduce uncertainty about their role in the workplace. This may exacerbate withdrawal tendencies (Lehman and Simpson, 1992; Levy, 2003; Petriglieri, 2011).

However, the effects of aging stereotype threat are not uniformly negative. Emerging research suggests that under certain conditions, individuals may resist and react against these stereotypes (Chiesa et al., 2019; Hoyt et al., 2010; Javadian and Zoogah, 2014; Schmader et al., 2008), leading to psychological reactance—a motivational state aimed at restoring threatened freedoms (Brehm and Brehm, 1981). This reactance may, in turn, fuel proactive work behaviors, where older workers actively seek to counteract stereotypes by engaging more fully in their work, adopting new challenges, and demonstrating their continued value to the organization (Crant, 2000; Griffin et al., 2007; Kray et al., 2002; Parker et al., 2006). The role of self-efficacy in this process cannot be understated; high levels of self-efficacy may amplify these reactions, with older workers in such conditions showing a stronger aging identity and reactance response to stereotype threat (Bandura, 1986).

**Figure 1:**
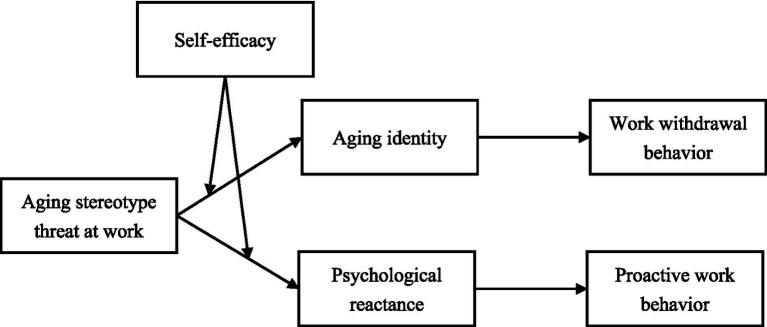
Overarching conceptual model.

This study seeks to fill the existing gap in the literature by exploring these dual pathways—how aging stereotype threat can lead both to work withdrawal and proactive work behaviors, incorporating self-efficacy as a moderator in this process (see Figure 1). By employing a dual mediation model grounded in Uncertainty-identity theory and psychological reactance theory, we investigate the roles of aging identity and psychological reactance in shaping these divergent outcomes. The findings from this research will not only contribute to a more nuanced understanding of aging stereotype threat, but also provide actionable insights for organizations aiming to foster an inclusive and supportive work environment for older employees (Kunze et al., 2015). By addressing both the negative and positive impacts of aging stereotype threat, this study aims to offer a comprehensive view of how older workers navigate the challenges posed by negative stereotypes and how organizations can better support them in doing so.

## Theory and hypotheses

### Aging stereotype threat

Aging refers to the progressive and degenerative changes in the structure and function of the human body that occur with advancing age. Stereotypes are generalized beliefs about the characteristics and behaviors typically attributed to specific groups. Everyone inevitably experiences aging and internalizes age-related stereotypes throughout his or her life (Levy, 2003). Aging stereotype threat specifically pertains to the societal beliefs and expectations held towards older adults (Levy et al., 1999). These stereotypes are not only held by younger individuals about older adults but are also internalized by older adults themselves (Hess et al., 2004; Levy, 2003; Remedios et al., 2010). Aging stereotypes encompass both positive and negative aspects. In the workplace, positive aging stereotypes characterize older workers as more reliable, loyal, enthusiastic, and possessing stronger social skills (Gringart et al., 2005; Gringart et al., 2012; Kluge and Krings, 2008; Shiu et al., 2015). Conversely, negative aging stereotypes depict older workers as less productive, with diminishing abilities to learn and use new technologies, and limited physical and mental capacities (Krings et al., 2011; McCann and Keaton, 2013). They are also often perceived as lacking initiative, being disinterested in learning and development, and resistant to change (Armstrong-Stassen and Schlosser, 2008; Maurer et al., 2008; Ng and Feldman, 2008, 2012).

When older workers become aware that they might be the targets of negative aging stereotypes, and they fear being judged or treated unfavorably based on these stereotypes, or worry that their performance in stereotype-relevant domains will confirm such negative stereotypes, they experience what is known as aging stereotype threat (Steele and Aronson, 1995). The concept of stereotype threat has been prevalent in social life, originating from early debates on whether the significant differences in academic performance between European-American and African-American individuals were due to genetic factors or the impact of negative stereotypes (Steele and Aronson, 1995). Research subsequently demonstrated that lower academic achievement among Black individuals, compared to their White counterparts, was a result of negative stereotypes rather than genetics. Stereotype threat places additional pressure on groups subjected to negative stereotypes, leading to underperformance in stereotype-relevant domains (Spencer et al., 2016). Regardless of the accuracy or validity of these stereotypes, their negative impact is evident (Block et al., 2011). While the content of aging stereotypes may vary across cultural contexts and time periods, negative stereotypes about aging generally outweigh positive ones (Dionigi, 2015). Belonging to a demographic minority group has been identified as a significant factor contributing to the experience of stereotype threat (Inzlicht and Ben-Zeev, 2000; Roberson et al., 2003). Therefore, it is crucial to study the impact of aging stereotype threat on older workers, particularly within workplace environments, where these individuals may be part of minority groups.

### Psychological responses to aging stereotype threat

Uncertainty-Identity Theory (U-I Theory) posits that individuals seek to reduce uncertainty by strengthening their identification with social groups, especially when their social identity is threatened or devalued (Hogg, 2012). In the context of aging stereotype threat, older workers who perceive that their age-related characteristics (such as declining productivity or resistance to change) are being negatively stereotyped may experience an increase in uncertainty about their own identity. To reduce this uncertainty, they may align more closely with the negative characteristics attributed to their age group, thereby reinforcing their aging identity. This process is particularly pronounced when the threat to one’s identity is perceived as uncontrollable or pervasive, prompting individuals to engage more deeply with their group identity (Hogg, 2007).

Aging identity refers to the self-perception of older individuals based on their association with the aging group and the evaluations this group receives from out-groups, such as beliefs in declining physical energy, work ability, responsibility, and motivation (Lamont et al., 2015). When older workers internalize the negative stereotypes associated with aging, this can strengthen their identification with the aging identity as a way to manage the uncertainty that comes from the stereotype threat. For instance, research on social stigma suggests that when individuals experience persistent negative judgments from others, they may internalize these judgments and increasingly identify with the stigmatized group, which can amplify the effects of stereotype threat (Corrigan et al., 2005). Therefore, the internalization of negative aging stereotypes can reinforce an individual’s aging identity, as they seek to resolve the uncertainty created by the threat (Chiesa et al., 2019). Therefore, we propose the following hypothesis.

*Hypothesis 1a:* Aging stereotype threat is positively correlated with aging identity.

While Uncertainty-Identity Theory (Hogg, 2012) explains that individuals facing identity threats may strengthen their group identity to reduce uncertainty, it also recognizes that not all individuals will passively accept negative stereotypes. Instead, individuals may seek to regain control over their social identity or to make their environment more predictable by actively resisting negative perceptions (Hogg, 2007; Swann et al., 2009). When individuals perceive that their identity is under threat, they may engage in behaviors aimed at counteracting or challenging the imposed stereotype, rather than internalizing it.

Psychological reactance theory (Brehm and Brehm, 1981) posits that when individuals perceive a threat to their freedoms—such as their freedom to maintain a positive self-concept—they experience a motivational drive to restore these freedoms. In the context of aging stereotype threat, older workers may perceive that their ability to maintain a positive self-identity is being undermined by external stereotypes. This perception can lead to psychological reactance, where individuals resist the imposed negative identity and actively seek to challenge and refute it (Dillard and Shen, 2005). For example, research has shown that individuals facing stereotype threats may engage in behaviors aimed at disproving the stereotypes, such as increased effort or the adoption of counter-stereotypical behaviors (Hoyt et al., 2010). This “I’ll show you” mentality is a manifestation of psychological reactance, reflecting a desire to regain control over one’s identity and how it is perceived by others (de Lemus et al., 2015). Therefore, we propose the following hypothesis:

*Hypothesis 1b:* Aging stereotype threat is positively correlated with psychological reactance.

### Aging identity as a linkage of aging stereotype threat and work withdrawal behavior

This study proposes that aging stereotype threat can lead work withdrawal behavior among older workers by reinforcing their aging identity. Work withdrawal behavior refers to the actions taken by employees to distance themselves from an unpleasant work environment when they perceive aversive situations within the organization (Rosse and Hulin, 1985; Gupta and Jenkins, 1991). These behaviors can be categorized into psychological withdrawal and behavioral withdrawal. Psychological withdrawal involves mentally disengaging from negative work situations, where employees remain physically present but are mentally detached (Lehman and Simpson, 1992). Common manifestations include daydreaming during work hours, discussing personal topics, and contemplating quitting. On the other hand, behavioral withdrawal encompasses actions aimed at physically distancing oneself from the unsatisfactory work environment, such as arriving late, being absent, taking frequent breaks, or even leaving the workplace entirely (Hanisch and Hulin, 1990).

Work withdrawal behavior can be understood as a progression, beginning with occasional daydreaming (psychological withdrawal), escalating to lateness and absenteeism (behavioral withdrawal), and potentially culminating in employee turnover (Gupta and Jenkins, 1991; Lehman and Simpson, 1992). Research on work withdrawal generally follows two approaches: “individual research,” which focuses on specific withdrawal behaviors (e.g., their antecedents and consequences), with turnover and absenteeism studies being particularly prominent; and “aggregated research,” which views various forms of withdrawal as part of a broader concept, suggesting that different withdrawal behaviors stem from similar antecedents. This study adopts the aggregated perspective to examine work withdrawal behavior.

Uncertainty-Identity Theory (Hogg, 2007) suggests that individuals’ attitudes and behaviors are influenced by their identity, especially when their group identity is threatened by negative stereotypes. When the self-concept is threatened, individuals may withdraw from their current identity to mitigate this threat (Petriglieri, 2011). For older workers, this could mean gradually dissociating their self-worth from areas associated with negative stereotypes, potentially resulting in work withdrawal behaviors (Steele et al., 2002; Nussbaum and Steele, 2007; Woodcock et al., 2012).

Older workers, who are often in the maintenance or decline stages of their careers, have limited mobility and fewer job options, making them more likely to continue working even in unsatisfactory environments (Gringart et al., 2013; Shacklock et al., 2007). According to Uncertainty-Identity Theory, when older workers encounter aging stereotype threat, they may remain in their jobs but adopt negative views of their group to protect their self-esteem, leading to psychological withdrawal, such as daydreaming. This psychological withdrawal often precedes behavioral withdrawal, manifesting as tardiness and absenteeism (Gupta and Jenkins, 1991; Lehman and Simpson, 1992). Some older workers might eventually opt for early retirement, influenced by the more positive stereotypes associated with retirees, such as being perceived as generous or sociable (McCann and Giles, 2002). For instance, studies have demonstrated that negative age stereotypes increase older workers’ intentions to retire (Weber et al., 2019).

Research on racial and gender stereotype threats has similarly found that people of color and women are more likely than white men to feel uncomfortable due to workplace discrimination, leading to withdrawal and turnover (Riordan et al., 2004; Roberson and Kulik, 2007). However, to date, no research has definitively confirmed the impact of aging identity, as influenced by aging stereotype threat, on work withdrawal behaviors. Thus, we propose the following hypotheses.

*Hypothesis 2:* Aging identity is positively correlated with work withdrawal behaviors.

*Hypothesis 3:* Aging identity mediates the relationship between aging stereotype threat and work withdrawal behaviors.

### Psychological reactance as a linkage of aging stereotype threat and proactive work behavior

This study proposes that aging stereotype threat can stimulate proactive work behavior among older workers by eliciting psychological reactance. Proactive work behavior refers to self-initiated, anticipatory actions aimed at improving the work environment (e.g., introducing new work methods, influencing organizational strategies) or enhancing one’s skills and abilities to meet future work demands (Crant, 2000; Griffin et al., 2007). The core attributes of proactive behavior are being future-focused (anticipating future challenges), change-oriented (actively seeking to effect change), and self-initiated (spontaneous, voluntary actions) (Parker et al., 2006). Proactive behaviors in the workplace can take many forms, such as adapting to new work conditions (Ashford and Black, 1996), voicing opinions (Van Dyne and LePine, 1998), initiating changes (Morrison and Phelps, 1999), expanding roles proactively (Parker et al., 1997), and solving problems or implementing new ideas (Parker et al., 2006). In today’s competitive and uncertain work environment, merely following managerial instructions is insufficient; it is increasingly critical for employees to exhibit proactivity (Grant and Ashford, 2008).

While extensive research has documented the negative consequences of stereotype threat, relatively few studies have explored its potential positive outcomes. Emerging evidence suggests that negative motivational states, such as those triggered by stereotype threat, can also drive proactivity. This is primarily because the change-oriented nature of proactive behavior enables individuals to shield themselves from unsatisfactory situations, particularly when they cannot easily exit the situation (Lebel, 2017). For instance, research has shown that negative emotions can motivate employees to address work-related challenges proactively (Bindl, 2019; Lebel, 2017).

According to Psychological Reactance Theory (Brehm and Brehm, 1981), when individuals experience reactance, they are motivated to restore the freedoms they perceive as threatened. This motivation often leads to a series of cognitive and behavioral efforts aimed at reclaiming their autonomy (Quick and Stephenson, 2008). The proactive motivation model suggests that having a “reason to” is a critical motivational state that drives proactive behavior (Parker et al., 2010). The psychological reactance state triggered by aging stereotype threat may motivate older workers to improve their circumstances and seek positive evaluations from their in-group, providing a compelling “reason” for proactive behavior. For example, Oyserman et al. (2001) found that when African Americans perceived racial bias, they worked harder to improve their math scores, countering negative stereotypes. Similarly, Heilman et al. (2004) discovered that women facing gender stereotype threat would adopt more masculine negotiation or communication strategies to enhance their performance, thereby refuting external stereotypes. In the context of aging stereotype threat, older workers might fear that their actions will confirm negative stereotypes, which limits their freedom to engage in work behaviors. In response, they may take proactive measures (e.g., actively participating in training, learning new skills) to improve their environment or enhance their capabilities, thus proving negative aging stereotypes wrong and restoring their behavioral freedom.

Uncertainty-Identity Theory (Hogg, 2007) suggests that when individuals face negative stereotypes related to their group, they may respond by strengthening their identification with the group in an attempt to reduce uncertainty. Aging stereotype threat makes older workers acutely aware of the disadvantaged position of both themselves and their group, which can lead to a rejection of these stereotypes and a determination to change their circumstances. Research on stereotype reactance indicates that when individuals encounter negative stereotype threats, they often engage in behaviors that directly contradict the stereotypes, a phenomenon known as reactance behavior (Kray et al., 2002; Kray et al., 2001). For example, older workers may increase their investment in their careers and work to counteract stereotypes related to diminished work enthusiasm due to age, or they may compete with younger colleagues to demonstrate that they are more capable (Desmette and Gaillard, 2008). Studies on racial and gender stereotype threats have also found that individuals might become more proactive at work to enhance their performance when confronted with stereotype threats (Kray et al., 2001).

Several influential theoretical perspectives emphasize the benefits of positive coping strategies, such as embracing challenges rather than succumbing to threats, adopting promotion-focused strategies rather than prevention-focused ones, and engaging in reactance resistance rather than accepting inequality and injustice (Blascovich and Tomaka, 1996; Higgins, 1998). This study hypothesizes that when older workers encounter aging stereotype threat, it may trigger psychological reactance, leading them to engage in proactive behaviors that can reverse or alter the negative identity of the aging group, thereby reducing their identity threat. Thus, we propose the following research hypotheses.

*Hypothesis 4:* Psychological reactance is positively correlated with proactive work behavior.

*Hypothesis 5:* Psychological reactance mediates the relationship between aging stereotype threat and proactive work behavior.

### The moderating role of self-efficacy

Self-efficacy refers to an individual’s belief in their ability to accomplish tasks and overcome challenges (Bandura, 1986). When faced with aging stereotype threat, older workers may experience heightened uncertainty regarding their identity and abilities. Uncertainty-Identity Theory (Hogg, 2012) suggests that individuals respond to threats to their identity by strengthening their identification with a group to reduce uncertainty. However, the strength of this response may vary depending on an individual’s self-efficacy.

Research on self-efficacy suggests that individuals with high levels of self-efficacy are more likely to engage in proactive behaviors and positively reinterpret negative situations (Bandura, 1986). In the context of aging stereotype threat, older workers with high self-efficacy may be more confident in their ability to overcome the negative effects of stereotypes and might therefore experience a stronger alignment with their aging identity as a way to combat stereotype threat. Conversely, older workers with low self-efficacy might feel less capable of countering negative stereotypes, leading to weaker identification with their aging identity. Thus, we hypothesize that:

*Hypothesis 6a:* Self-efficacy moderates the relationship between aging stereotype threat and aging identity, such that the relationship is stronger for older workers with higher levels of self-efficacy than for those with lower levels of self-efficacy.

Self-efficacy not only influences identity processes but also shapes how individuals respond to threats to their autonomy and freedom. According to Psychological Reactance Theory (Brehm and Brehm, 1981), when individuals perceive a threat to their freedom, including their ability to maintain a positive self-concept, they experience psychological reactance—a motivational drive to restore their perceived freedoms. In the context of aging stereotype threat, older workers may feel that their ability to act competently is being undermined. The intensity of their psychological reactance may be influenced by their self-efficacy, as it modulates their belief in their ability to resist such threats.

Research by Kray et al. (2004) suggests that individuals are more likely to successfully react to stereotypes when they have sufficient power or ability. This implies that older workers with high self-efficacy may be more motivated to take proactive actions to counteract aging stereotype threats, engaging in behaviors that restore their autonomy and challenge negative stereotypes. Conversely, those with low self-efficacy may feel less capable of resisting stereotype threats, leading to weaker psychological reactance.

Further, Hoyt and Blascovich (2007) demonstrated that women with sufficient self-efficacy exhibited stronger reactance when confronted with gender stereotypes, highlighting the critical role self-efficacy plays in responses to stereotype threats. In the case of aging stereotype threat, older workers with high self-efficacy may be more likely to engage in proactive behaviors (such as seeking training or demonstrating new skills) to counteract the stereotype and reinforce their perceived competence. By contrast, older workers with low self-efficacy may feel powerless to challenge the stereotype, resulting in weaker reactance. Therefore, we hypothesize:

*Hypothesis 6b:* Self-efficacy moderates the relationship between aging stereotype threat and psychological reactance, such that the relationship is stronger for older workers with higher levels of self-efficacy than for those with lower levels of self-efficacy.

## Method

### Participants and procedures

In order to be eligible to participate in this study, participants were required to (1) be employed full-time, (2) be above 40 years old, and (3) work in an organizational setting. This study used a two-wave study design (over a month period). specifically, we measured demographic variables (e.g., age, gender), self-efficacy, aging stereotype threat, aging identity, and psychological reactance at time 1. At time 2, work withdrawal behavior and proactive work behavior were measured.

The data for this study were collected via a combination of convenience and snowball sampling. Eighty undergraduate student volunteers in psychology classes were trained by research assistants. Then these student volunteers approached and introduced the study information to potential respondents. If potential participants expressed an interest in this study, and met the inclusion criteria, they were sent an online survey link through a smartphone application (i.e., WeChat or QQ, commonly used social media in China). After instructing respondents how to participate, each of them was assigned a code which was used to match their responses across two times. All participants were informed that their responses were confidential and only the researchers can access to the data. IRB approval from the researchers’ affiliated institution was obtained for the data collection. Each participant who successfully completed his or her survey was rewarded with ¥10 (about $ 1.5) at time 1, and ¥15 (about $ 2) at time 2. Each student volunteer who helped to recruit participants was given extra credit points in a course in psychometrics and rewarded with ¥10 (about $1.5) for each survey completed by the participant they have successfully recruited.

At time 1, 610 employees who met the study criteria were invited for the time 1 survey. 552 (90%) completed surveys were acquired at time 1. Among these 552 respondents, 441 (80%) completed the survey at time 2. The final sample consisted of 441 older workers from a variety of industries, with an average age of 48.63 (SD = 3.66). There were 231 males (52%) and 210 females (48%). 189 participants (43%) had education below university level, and 252 participants (57%) had a university degree or higher. 197 participants (45%) were front-line employees, 121 (27%) were front-line managers, 101 (23%) were middle managers, and 22 (5%) were senior managers.

### Measurement

All the scales were originally in English and then translated into Chinese by the researchers who are fluent in both languages using back-translation methods (Brislin, 1970).

#### Aging stereotype threat

Aging stereotype threat was measured using the 5-item scale developed by Roberson et al. (2003). Sample items are “In my company, some people think I am less competent because of my age” and “In my company, some people think people of my age have difficulty passing performance evaluations.” Participants indicated their agreement with each statement on a 5-point Likert scale, ranging from 1 (*strongly disagree*) to 5 (*strongly agree*). The Cronbach’s alpha for the total scale was 0.91.

#### Self-efficacy

The 10-item self-efficacy scale developed by Schwarzer et al. (1997) was used. Sample items are “I can always manage to solve difficult problems if I try hard enough,” “It is easy for me to stick to my aims and accomplish my goals” and “No matter what comes my way, I’m usually able to handle it.” Participants indicated their agreement with each statement on a 5-point Likert scale, ranging from 1 (*strongly disagree*) to 5 (*strongly agree*). The Cronbach’s alpha for the self-efficacy scale was 0.93.

#### Aging identity

The 12-item aging identity scale developed by Tougas et al. (2004) was used. Sample items are “My work efficiency was low,” “I found it hard to adapt to changes” and “I found it difficult to learn new things.” Participants indicated their agreement with each statement on a 5-point Likert scale, ranging from 1 (*strongly disagree*) to 5 (*strongly agree*). The Cronbach’s alpha for the total scale was 0.95.

#### Psychological reactance

Psychological reactance is a concept encompassing both state and trait dimensions (Rains, 2013). While a substantial body of research has conceptualized it as a stable personality trait (e.g., Hong and Faedda, 1996), some studies have also treated it as a state variable elicited by specific situational triggers. In this study, we operationalized psychological reactance as a state-specific response to aging stereotype threat. Given the lack of existing measures directly assessing the psychological reactance of older workers in such contexts, we developed a 5-item scale based on prior research on state reactance (e.g., Dillard and Shen, 2005). The scale items included statements such as, “At work, when someone thinks employees my age are incompetent, I feel compelled to refute them,” “I feel annoyed when someone doubts my competence because of my age,” “When someone believes that employees my age struggle to pass performance evaluations, I work hard to prove them wrong,” “I feel upset when someone in the workplace makes biased evaluations of employees my age,” and “When my supervisor assumes that I will perform poorly because of my age, I take action to refute them.” Participants rated their agreement on a 5-point Likert scale, ranging from 1 (strongly disagree) to 5 (strongly agree). This scale captures momentary reactions to age-related stereotypes, reflecting how individuals react when they perceive their competence being questioned due to their age. Exploratory factor analysis (EFA) using SPSS 24.0 confirmed the scale’s unidimensionality. The Kaiser–Meyer–Olkin (KMO) value was 0.91, and Bartlett’s test of sphericity was significant (*χ^2^* = 1088.98, *df* = 10, *p* < 0.01). A single factor emerged, with factor loadings ranging from 0.78 to 0.84, explaining 66.61% of the total variance. The Cronbach’s alpha for the scale was 0.87, indicating good internal consistency.

#### Work withdrawal behavior

Work withdrawal behavior was measured using the 12-item scale developed by Lehman and Simpson (1992). The 12-item scale consists of two dimensions: psychological withdrawal (8 items, e.g., and “I have thoughts of being absent from work” and “I daydream at work”) and behavioral withdrawal (4 items, e.g., “I sleep during work hours” and “I leave my post without permission”). Participants rated each item on a 5-point scale (1 = strongly disagree, 5 = strongly agree). The Cronbach’s alpha for the total scale was 0.93.

#### Proactive work behavior

Proactive work behavior was self-rated using the individual task proactivity (3 items, e.g., “Initiated better ways of doing your core tasks”), team member proactivity (3 items, e.g., “Developed new and improved methods to help your work unit perform better”), and organization member proactivity (3 items, e.g., “Made suggestions to improve the overall effectiveness of the organization”) subscales developed by Griffin et al. (2007). In accordance with other research (Ma and Peng, 2019), we combined the three aspects to better gauge the overall proactive work behavior an employee can display at work. Participants were instructed to report how often they have carried out each of the behavior over the past month on a scale ranging from 1 (never) to 5 (most of the time). The Cronbach’s alpha for the total scale was 0.93.

##### Control variables

To avoid potential confounding effects, we controlled for the participants’ *gender*, *age*, and *level of education*.

### Analytical approach

We performed structural equation modeling (SEM) analyses using AMOS 22.0 to test our hypotheses. To maximize degrees of freedom and to avoid misidentification of latent variable structural models, we constructed item parcels for measures consisting of five or more items. We utilized the approach suggested by Little et al. (2002), which involves balancing the best and worst items (based on the value of factor loadings obtained from Exploratory Factor Analysis) across the parcels, and formed three parcels each for aging stereotype threat, self-efficacy, aging identity, psychological reactance, work withdrawal behavior and proactive work behavior, respectively. Latent variable such as proactive work behavior was operationalized by aggregating dimensions. The latent interaction variable was created using the product-indicator approach as recommended by Marsh et al. (2004). Specifically, we first standardized the item parcels of both predictors—aging stereotype threat and self-efficacy—to ensure comparability and reduce multicollinearity. Each parcel of aging stereotype threat was then multiplied with the corresponding parcel of self-efficacy to generate interaction terms. These product terms served as indicators for the latent interaction variable. This approach allows for the proper estimation of interaction effects in structural equation modeling (SEM), maintaining the integrity of the latent constructs while enabling the examination of moderation relationships.

In order to test our hypotheses, we followed Anderson and Gerbing (1988) two-step approach for structural equation modeling (SEM) using AMOS 22.0. In the first step, we constructed a baseline model incorporating all variables and structural paths except self-efficacy and the interaction effect. This model included the main effects of aging stereotype threat on aging identity and psychological reactance. To compare the baseline model with the following nested hypothesized model, we constrained the paths from self-efficacy to aging identity and psychological reactance to zero. We also constrained the paths from the interaction term, aging stereotype threat × self-efficacy, to aging identity and psychological reactance to zero. Next, in addition to the baseline model’s paths, we introduced the effects from self-efficacy to aging identity and psychological reactance, as well as the effects of the interaction latent variable to aging identity and psychological reactance in our hypothesized model. Following the suggestion of Preacher and colleagues’ (Preacher et al., 2007), an empirical bootstrapping approach with 95% bias-corrected confidence intervals (CIs) was used to test for the significance of indirect effects in the final selected model.

## Results

### Preliminary analyses

Before testing our hypotheses, confirmatory factor analyses (CFA) were conducted to assess the discriminant and convergent validity of all study variables. The results of CFA of the six-factor model [*χ^2^*(120) =218.87, *χ^2^*/*df* = 1.82, root mean square error of approximation (RMSEA) = 0.04, Bollen’s incremental fit index (IFI) = 0.98, Comparative Fit Index (CFI) = 0.98, Tucker-Lewis Index (TLI) = 0.98] met Hu and Bentler (1999) conservative two-index presentation criteria for good model fit. Alternative models were also compared, indicating that the six-factor model fits the data considerably better than did any of the alternative models, thus supporting the discriminant validity of the measures (see Table 1).

**Table 1:** The results of confirmatory factor analyses.

Models	$\chi^{2}$	*df*	$\chi^{2}/df$	CFI	TLI	IFI	RMSEA
Six-factor model	218.87	120	1.82	0.98	0.98	0.98	0.04
Five-factor model	1140.64	125	9.13	0.83	0.80	0.83	0.14
Four-factor model	2081.51	129	16.14	0.68	0.62	0.68	0.18
Three-factor model	2719.74	132	20.60	0.58	0.51	0.58	0.21
Two-factor model	3563.32	134	26.59	0.44	0.36	0.44	0.24
One-factor model	4207.68	135	31.17	0.33	0.24	0.33	0.26

One-factor model = self-efficacy + aging stereotype threat + aging identity + psychological reactance + work withdrawal behavior + proactive work behavior); Two-factor model = self-efficacy + aging stereotype threat + aging identity + psychological reactance, work withdrawal behavior + proactive work behavior; Three-factor model = self-efficacy + aging stereotype threat + aging identity + psychological reactance, work withdrawal behavior, proactive work behavior; Four-factor model = self-efficacy + aging stereotype threat, aging identity + psychological reactance, work withdrawal behavior, proactive work behavior; Five-factor model = self-efficacy + aging stereotype threat, aging identity, psychological reactance, work withdrawal behavior, proactive work behavior; Six-factor model = self-efficacy, aging stereotype threat, aging identity, psychological reactance, work withdrawal behavior, proactive work behavior.

Table 2 provides means, standard deviations, and correlations among all study variables. As reported, the pattern of bivariate relationships was in the expected direction. Aging stereotype threat was positively related to employees’ aging identity (*r* = 0.46, *p* < 0.01) and psychological reactance (*r* = 0.10, *p* < 0.05). Aging identity was positively related to work withdrawal behavior (*r* = 0.53, *p* < 0.01). Psychological reactance was positively related to proactive work behavior (*r* = 0.25, *p* < 0.01). Self-efficacy was negatively related to employees’ aging identity (*r* = −0.23, *p* < 0.01) and positively related to psychological reactance (*r* = 0.31, *p* < 0.01).

**Table 2:** Mean, standard deviations and correlations of study variables (*N* = 441).

Variables	M	SD	1	2	3	4	5	6	7	8
1. Gender (T1)	0.52	0.50								
2. Age (T1)	48.63	3.66	0.20**							
3. Education (T1)	2.62	1.22	0.08	−0.11*						
4. Aging stereotype threat (T1)	2.78	0.99	−0.01	−0.11*	−0.11*					
5. Self-efficacy (T1)	3.82	0.65	0.04	0.01	0.05	−0.08				
6. Aging identity (T1)	2.36	0.87	−0.00	0.09*	−0.18*	0.46**	−0.23**			
7. Psychological reactance (T1)	3.48	0.84	0.04	−0.00	−0.00	0.10*	0.31**	0.14**		
8. Work withdrawal behavior (T2)	2.23	0.87	0.06	0.06	−0.03	0.21**	−0.24**	0.53**	−0.01	
9. Proactive work behavior (T2)	4.00	0.79	−0.00	0.01	0.16**	−0.11*	0.49**	−0.31**	0.25**	−0.24**

T1 = Time 1, T2 = Time 2; * *p* < 0.05, ** *p* < 0.01.

### Hypothesis testing

**Figure 2:**
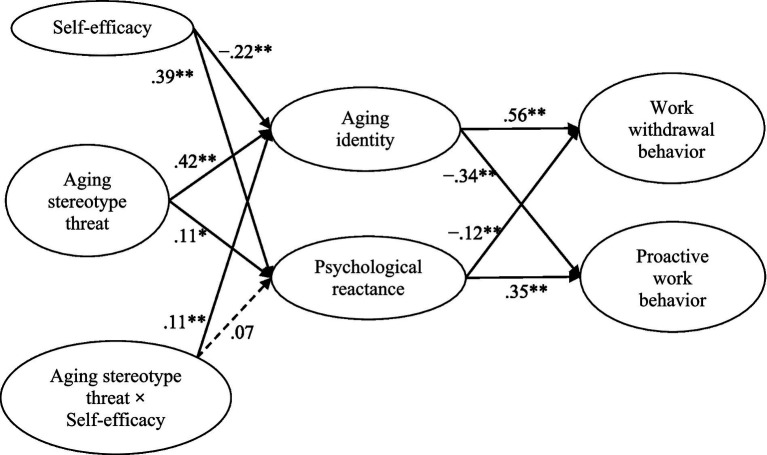
Structural equation modeling results for the hypothesized model. Values shown are standardized parameter estimates. Solid lines are significant, dashed lines are not significant. Controlling for gender, age, level of education and level of position is not presented for the sake of clarity. ** *p* < 0.01, * *p* < 0.05.

Hypotheses were tested using AMOS 22.0. In the structural equation modeling analysis below, model comparison was conducted between the mediated model with control variables, excluding the interaction effects of aging stereotype threat and self-efficacy (Model 1, with all the paths from control variables, i.e., gender, age and education, the effects from self-efficacy, and the interaction effects of aging stereotype threat and self-efficacy were constrained to be zero) and the hypothesized mediated model with control variables, self-efficacy and the interaction effects (Model 2). The results showed that the hypothesized model fit the data well (Model 2, *χ^2^* (230) = 485.740, *χ^2^*/*df* = 2.112, *p* < 0.001; RMSEA = 0.05, CFI = 0.96, IFI = 0.96, TLI = 0.96), and the change in chi-square test showed that the fit was significantly better than Model 1, which was the mediated model without the interaction effects of aging stereotype threat and self-efficacy (Model 1, *χ^2^* (234) = 575.497, *χ^2^*/*df* = 2.459, *p* < 0.001; RMSEA = 0.06, CFI = 0.95, IFI = 0.95, TLI = 0.94) (Δ*x^2^ =* 89.757, Δ*df* = 4, *p* < 0.01). We then tested the partially mediated model with control variables and the interaction effects of aging stereotype threat and self-efficacy, including the direct paths from aging stereotype to work withdrawal behavior and proactive work behavior (Model 3). The result showed that Model 3 fitted the data well (for Model 3, *χ^2^* (228) = 484.173, *χ^2^*/*df* = 2.124, *p* < 0.001; RMSEA = 0.05, CFI = 0.96, IFI = 0.96, TLI = 0.95); however, the direct paths form aging stereotype threat to work withdrawal behavior (*β* = −0.06, *p* > 0.05, *ns*; CI95% = [−0.16, 0.06]) and proactive work behavior (*β* = 0.04, *p* > 0.05, *ns*; CI95% = [−0.08, 0.15]) were insignificant. Thus, we concentrated our analyses on the Model 2 (see Figure 2).

The results based on Model 2 are shown in Figure 2. Hypotheses 1a was supported, as aging stereotype threat was positively related to aging identity (*β* = 0.42, *p* < 0.01; CI95% = [0.33, 0.51]). Aging identity was positively associated with work withdrawal behavior (*β* = 0.56, *p* < 0.01; CI95% = [0.45, 0.65]), supporting Hypothesis 2. Bootstrapping analyses showed that the indirect effect of aging stereotype threat on work withdrawal behavior through aging identity was significant (effect =0.21, SE = 0.03; CI95% = [0.15, 0.28]), supporting Hypothesis 3. Additionally, aging stereotype threat was positively related to psychological reactance (*β* = 0.11, *p* < 0.05; CI95% = [0.01, 0.22]), supporting Hypothesis 1b. Psychological reactance was positively associated with proactive work behavior (*β* = 0.35, *p* < 0.01; CI95% = [0.23, 0.46]), lending support for Hypothesis 4. Bootstrapping analyses showed that the indirect effect of aging stereotype threat on proactive work behavior through psychological reactance was significant (effect = 0.02, SE = 0.01; CI95% = [0.01, 0.05]), supporting Hypothesis 5.

**Figure 3:**
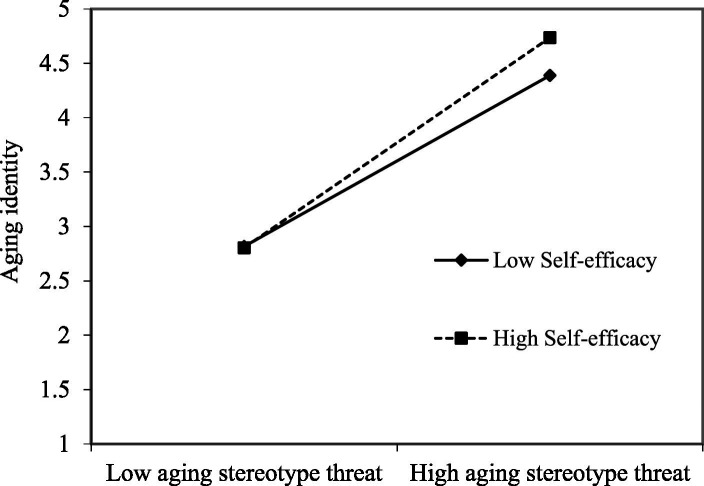
The relationship between aging stereotype threat and aging identity moderated by self-efficacy.

As illustrated in Figure 2, self-efficacy was negatively associated with aging identity (*β* = −0.22, *p* < 0.01; CI95% = [−0.33, −0.10]). A significant interaction term between aging stereotype threat and self-efficacy predicted aging identity (*β* = 0.11, *p* < 0.05; CI95% = [0.01, 0.22]). Figure 3 illustrates this moderation effect (Aiken and West, 1991). A simple slope test showed that the relationship between aging stereotype threat and aging identity was significantly positive at a high level of self-efficacy (1SD above the mean, simple slope = 0.49, *p* < 0.01), but not at a low level of self-efficacy (1SD below the mean, simple slope = 0.21, *p* > 0.05, *ns*). Therefore, Hypothesis 6a was supported. Although self-efficacy was positively related to psychological reactance (*β* = 0.39, *p* < 0.01; CI95% = [0.26, 0.52]) in Figure 2, the interaction term between aging stereotype threat and self-efficacy did not significantly affect psychological reactance (*β* = 0.07, *p* > 0.05, *ns*; CI95% = [−0.09, 0.22]). Thus, Hypothesis 6b was not supported.

### Additional analyses

The findings in Figure 2 show that aging identity is negatively associated with proactive work behavior (*β* = −0.32, *p* < 0.01; CI95% = [−0.42, −0.25]), and psychological reactance is negatively related to work withdrawal behavior (*β* = −0.12, *p* < 0.01; CI95% = [−0.23, −0.02]). To assess the conditional indirect effects of aging stereotype threat, we followed the approach outlined by Edwards and Lambert (2007) and calculated these effects at low and high levels of self-efficacy (i.e., +/− 1 SD from the mean). The conditional indirect effects showed that for employees with low self-efficacy, the indirect effect of aging stereotype threat on work withdrawal behavior through aging identity was significant (indirect effect = 0.16, SE = 0.03, 95% CI = [0.09, 0.23]). Similarly, the indirect effect was also significant for employees with high self-efficacy (indirect effect = 0.25, SE = 0.04, 95% CI = [0.17, 0.33]). The difference between the indirect effects at high and low levels of self-efficacy was also significant for work withdrawal behavior (difference = 0.09, 95% CI = [0.08, 0.11]). These results indicate that the indirect effect of aging stereotype threat on work withdrawal behavior through aging identity is significant, with the effect being stronger for employees with higher self-efficacy.

## Discussion

This study examines how aging stereotype threat influences older workers’ behaviors through a dual mediation model, guided by Uncertainty-Identity Theory and Psychological Reactance Theory. The findings reveal that aging stereotype threat significantly strengthens aging identity, which in turn leads to work withdrawal behaviors, with aging identity fully mediating this relationship. Moreover, aging stereotype threat triggers psychological reactance, promoting proactive work behaviors as older workers attempt to counteract negative stereotypes and restore their self-esteem. Additionally, the results show that aging identity not only drives work withdrawal behaviors but also decreases proactive behaviors, an unintended consequence. On the other hand, psychological reactance reduces work withdrawal behaviors while promoting proactive efforts to challenge stereotypes. The study also finds that self-efficacy moderates the relationship between aging stereotype threat and aging identity, with stronger effects for those with higher self-efficacy. These results highlight the complex and dual nature of aging stereotype threat, emphasizing the need for interventions to address both its negative and positive effects on older workers’ behavior.

### Theoretical implications

The present study makes several theoretical contributions. First, while aging stereotypes are well-documented in the literature (Kulik et al., 2016), there has been limited research specifically examining aging stereotype threat in the workplace, especially in relation to its dual positive and negative effects. Unlike previous studies that focus solely on the detrimental effects of stereotype threat, this study is the first to simultaneously explore both the negative (e.g., aging identity and work withdrawal behavior) and positive (e.g., proactive work behavior) outcomes of aging stereotype threat, highlighting its “double-edged sword” effect. This approach broadens our understanding of the potential consequences of stereotype threat and provides a foundation for future studies exploring the positive effects of age-based stereotype threat. Additionally, this study responds to previous calls for expanding research on the consequences of aging stereotype threat (Kulik et al., 2016), specifically its impact on older employees’ work behaviors.

Second, this study applies Uncertainty-Identity Theory and Psychological Reactance Theory to explore the psychological mechanisms through which aging stereotype threat influences work behaviors. Over the past decade, industrial and organizational psychology has increasingly adopted the social identity approach to investigate older adults’ experiences in the workplace (e.g., Desmette and Gaillard, 2008; Hesketh et al., 2015). This research suggests that social identity processes significantly affect older employees’ cognition, emotions, motivation, attitudes, and behaviors (Hornsey, 2008). Uncertainty-Identity Theory posits that individuals respond to threats to their identity by strengthening their group identification to reduce uncertainty (Hogg, 2007). However, few studies have directly explored the impact of aging stereotype threat through this lens. This study extends the literature by demonstrating that, in addition to reinforcing aging identity, aging stereotype threat may also induce psychological reactance—a motivational drive to restore the freedom and autonomy perceived as being threatened. By combining social identity and psychological reactance perspectives, this study provides a more nuanced understanding of how stereotype threats impact self-identity and work behaviors. Importantly, while aging identity was positively correlated with work withdrawal behaviors, and psychological reactance with proactive work behaviors, the study also found that aging identity was negatively related to proactive work behaviors, and psychological reactance was negatively related to work withdrawal behaviors. The findings highlight the complex psychological mechanisms linking aging stereotype threat to varied work behaviors.

Third, this study contributes to the literature on work withdrawal behaviors among older workers. Work withdrawal behaviors, including absenteeism, turnover, and reduced effort (Hanisch and Hulin, 1990), have significant implications for organizations, especially in light of the aging workforce. Despite the importance of understanding these behaviors, there has been limited research linking aging stereotype threat to work withdrawal. Although some studies have briefly discussed how stereotype threat might lead to disengagement (Woodcock et al., 2012), this study is the first to directly examine how aging stereotype threat impacts work withdrawal behaviors, filling an important gap in the literature and expanding our understanding of withdrawal behaviors in the context of aging.

Fourth, this study contributes to the literature on the proactivity of older workers. Previous scholars have often emphasized that positive motivational states promote proactive work behaviors (Parker et al., 2010). However, increasing attention is being paid to how negative motivational states and work stressors can also enhance proactivity (Fritz and Sonnentag, 2009). Considering that proactivity is crucial for successful aging at work, exploring the antecedents of proactive behavior among older workers is a valuable research direction (Zacher, 2015; Zacher and Kooij, 2017). However, there remains limited understanding of the factors that trigger proactivity among older workers, particularly whether negative motivators (such as negative experiences at work) can induce proactivity. This study demonstrates that aging stereotype threat can induce psychological reactance, leading to proactive work behaviors, albeit to a lesser degree than the negative effects. This finding suggests that individuals who experience momentary reactions to stereotype threat are more likely to engage in behaviors that counteract age-related stereotypes. By conceptualizing psychological reactance as a state response rather than a trait, we capture the immediate emotional and cognitive reactions of individuals when they perceive their competence being questioned due to age-related stereotypes in the workplace. This finding also broadens our understanding of how negative motivators can lead to proactivity in the workplace.

Finally, an important aspect of this study was the inclusion of self-efficacy as a moderator in the relationship between aging stereotype threat and both aging identity and psychological reactance. The results show that higher levels of self-efficacy strengthen the relationship between aging stereotype threat and aging identity, suggesting that older workers with greater self-efficacy are more likely to counter stereotype threats by reinforcing their identification with the aging group. However, this also may lead to more work withdrawal behaviors and less proactive work behaviors. This finding challenges the conventional assumption that increasing self-efficacy will always reduce negative outcomes. This finding underscores the complex role of self-efficacy in shaping responses to identity threats in the workplace.

While most of our hypotheses were supported, the moderating effect of self-efficacy on the relationship between aging stereotype threat and psychological reactance was not significant. This finding raises important theoretical questions. Prior research has consistently shown that self-efficacy influences how individuals respond to stereotype threats (Hoyt and Blascovich, 2007; Kray et al., 2004), yet in the context of aging stereotype threat, our results suggest that self-efficacy may not significantly moderate this relationship. One possible explanation is that, for older workers, factors such as accumulated work experience or external support systems might play a more prominent role in shaping their reactions to stereotype threats. Alternatively, the effect of self-efficacy may be diminished in the case of aging stereotypes, which evoke deeply ingrained, identity-related concerns that are less flexible or amenable to change compared to other types of stereotype threats. Thus, while self-efficacy has proven influential in other contexts, its role in mitigating the effects of aging stereotype threat appears to be more complex and context-dependent. Future research should investigate other moderating factors, such as workplace support systems and organizational culture, to better understand how older workers navigate stereotype threats and how these factors may interact with self-efficacy.

### Practical implications

This study has important practical implications for organizations and managers. First, it highlights the need for managers to recognize the impact of aging stereotype threat on older workers’ behaviors. The results show that aging stereotype threat significantly influences work withdrawal behaviors, which can be costly for organizations and set a negative example (Mathieu and Kohler, 1990). Therefore, organizations should proactively address negative aging stereotypes. While older workers’ experience and expertise are invaluable, they are often hindered by these stereotypes. To reduce negative perceptions, organizations should implement age-inclusive human resource management (HRM) practices, which have been shown to reduce age stereotypes (Kunze et al., 2015). For example, mentorship programs and intergenerational collaboration can promote knowledge-sharing and foster an inclusive work culture (Abrams et al., 2006). Additionally, managers should overcome biases and learn to value the diverse skills that older employees bring to the workplace.

Second, this study underscores the importance of aging identity in the relationship between aging stereotype threat and work withdrawal behaviors. Addressing identity issues among older workers is essential. Research suggests that individuals often view themselves through the lens of their most stigmatized identity (Kulik, 2014). Organizations can help by creating a supportive environment where older workers are respected and their value is recognized. Assigning roles that allow them to showcase their abilities can enhance job engagement and foster a positive self-perception. It is also important for older workers to actively adjust to their circumstances, recognize their strengths, and maintain a positive identity despite aging stereotype threats.

Third, the study found that aging stereotype threat can trigger psychological reactance, which can lead to proactive work behaviors. Organizations can harness this reactance to foster positive outcomes, such as challenging stereotypes and encouraging older workers to demonstrate their competence. However, it is important to manage this reactance carefully, as striving to disprove stereotypes can sometimes result in psychological costs (Block et al., 2011). Therefore, organizations should monitor the psychological well-being of older workers and ensure that the drive to counteract stereotypes does not lead to burnout or stress.

Lastly, the study suggests that, for older workers with higher self-efficacy, aging stereotype threats may reinforce their aging identity, which could trigger more withdrawal behaviors rather than reduce them. Therefore, while organizations should continue to foster self-efficacy in older workers, they should also be mindful of the potential unintended consequences of higher self-efficacy in the context of aging stereotype threats. Specifically, organizations should focus on creating environments that mitigate the negative impact of aging stereotypes and provide supportive resources for older workers to help them navigate stereotype-related challenges effectively.

### Limitations and future research directions

While this study provides valuable theoretical and practical insights, it has several limitations. First, the cross-sectional design limits our ability to draw causal conclusions. This study primarily examined the unidirectional impact of aging stereotype threat on older workers’ identity and behaviors. However, these variables may have reciprocal effects over time. For example, societal ageism could lead older individuals to internalize an aging identity, which could reinforce societal stereotypes, creating a vicious cycle. Future studies should use longitudinal or experimental designs to examine causal and reciprocal relationships.

Second, the reliance on self-report measures raises concerns about common method variance. To mitigate this, we used a two-wave design over a one-month period and followed Podsakoff et al. (2003) and Williams and McGonagle (2016) recommendations to reduce common method bias. We also tested for common method variance by controlling for the effects of an unmeasured latent method factor (ULMC) in our confirmatory factor analysis. The results showed no significant improvement in model fit when comparing a seven-factor model (with a common method factor) to the six-factor model, suggesting that common method bias is not a significant concern in this study (Chen, 2007).

Third, while this study examined the moderating role of self-efficacy in the relationship between aging stereotype threat and aging identity, future research could further explore the boundary conditions of this moderation. Specifically, our results revealed that higher self-efficacy strengthens the relationship between aging stereotype threat and aging identity, but it also leads to more work withdrawal behaviors for older workers with higher self-efficacy. This unexpected finding suggests that self-efficacy does not always produce a positive outcome in the context of aging stereotype threat. Therefore, it would be valuable to investigate moderators that could influence the effects of aging stereotype threat, such as the workplace environment, organizational support, social comparison or personal resources. Future research could explore when and why self-efficacy might mitigate or exacerbate the negative effects of aging stereotype threat on older workers’ behaviors. Understanding these dynamics will provide a more nuanced view of the role of self-efficacy in shaping responses to stereotype threats.

Fourth, in this study, we developed a 5-item psychological reactance scale to measure the state of reactance in older workers facing aging stereotype threat. However, we acknowledge that this self-developed tool requires further validation. Future research should focus on examining the reliability and validity of this scale to ensure its robustness. Additionally, it would be valuable to explore whether trait-based psychological reactance moderates the relationship between aging stereotype threat and aging identity, which could provide further insights into how individual differences influence reactions to stereotype threats in the workplace.

Finally, we chose age 40 as the threshold for inclusion (the mean age of our sample is 48.63, and SD = 3.66), as it marks the onset of aging stereotype threat in the workplace. However, the relatively younger age distribution may limit the study’s ability to capture the experiences of workers closer to statutory retirement age in China, particularly between 55 and 60. Future research should extend the age range to include these older employees to better understand how aging stereotype threat affects workers approaching retirement. Additionally, our study is context-specific to China, where the average retirement age is much earlier (around 55) than in Western countries (around 65). This unique retirement system and age discrimination in the workplace may limit the generalizability of our findings to other countries with different retirement systems. Therefore, future research should investigate aging stereotype threat across various countries and retirement systems to enhance the applicability of the results.

## Data Availability

The original contributions presented in the study are included in the article/supplementary material, further inquiries can be directed to the corresponding author.
